# Ovarian Cancer and *BRCA1/2* Testing: Opportunities to Improve Clinical Care and Disease Prevention

**DOI:** 10.3389/fonc.2016.00119

**Published:** 2016-05-11

**Authors:** Katherine Karakasis, Julia V. Burnier, Valerie Bowering, Amit M. Oza, Stephanie Lheureux

**Affiliations:** ^1^Drug Development Program, Division of Medical Oncology and Hematology, Princess Margaret Cancer Centre, University of Toronto, Toronto, ON, Canada

**Keywords:** ovarian cancer, *BRCA1/2*, testing, treatment, prevention

## Abstract

Without prevention or screening options available, ovarian cancer is the most lethal malignancy of the female reproductive tract. High-grade serous ovarian cancer (HGSOC) is the most common histologic subtype, and the role of germline *BRCA1/2* mutation in predisposition and prognosis is established. Given the targeted treatment opportunities with PARP inhibitors, a predictive role for *BRCA1/2* mutation has emerged. Despite recommendations to provide *BRCA1/2* testing to all women with histologically confirmed HGSOC, uniform implementation remains challenging. The opportunity to review and revise genetic screening and testing practices will identify opportunities, where universal adoption of *BRCA1/2* mutation testing will impact and improve treatment of women with ovarian cancer. Improving education and awareness of genetic testing for women with cancer, as well as the broader general community, will help focus much-needed attention on opportunities to advance prevention and screening programs in ovarian cancer. This is imperative not only for women with cancer and those at risk of developing cancer but also for their first-degree relatives. In addition, *BRCA1/2* testing may have direct implications for patients with other types of cancers, many of which are now being found to have *BRCA1/2* involvement.

## Introduction

**Figure 1:**
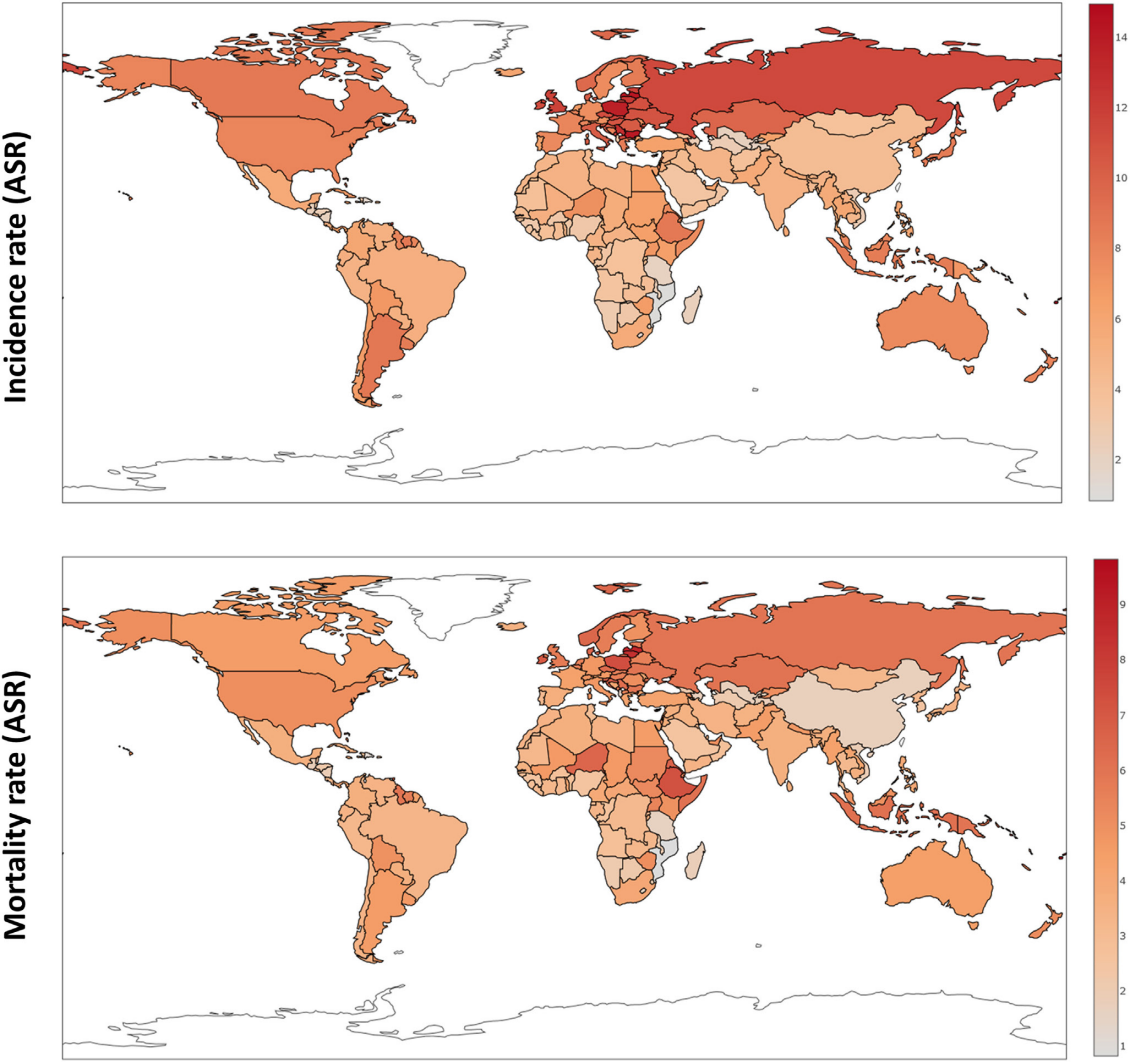
**Ovarian cancer incidence and mortality rates worldwide (ASR) based on GloboCan data (1)**.

Over the last four decades, there has been modest progress in the 5-year overall survival rates of women diagnosed with ovarian cancer, despite enhanced surgical efforts and introduction of doublet platinum/taxane chemotherapy. Worldwide, newly diagnosed cases of ovarian cancer have reached 239,000, positioning this malignancy as the seventh most common cancer in all women, with the highest incidence in Europe and North America (1). Typically diagnosed at an advanced stage (III/IV), high mortality rates for ovarian cancer continue to persist with almost 152,000 deaths per year (Figure 1) (2).

The lifetime risk of spontaneously developing and dying from ovarian cancer are 1.39 and 1.04%, respectively; however, the incidence of developing ovarian cancer significantly increases in carriers of germline mutations, mainly with either the *breast cancer gene 1* (*BRCA1*) or *2* (*BRCA2*) genes. The lifetime risk of developing ovarian cancer is 40–60 and 11–27% for *BRCA1* and *BRCA2* mutation carriers, respectively (3). These particular mutations are implicated in 10–15% of all ovarian cancer cases and almost 20% of high-grade serous histology [high-grade serous ovarian cancer (HGSOC)] (4), including in women without a family history of breast or ovarian cancer. Approximately, one-third of patients with hereditary ovarian cancer have no close relatives with cancer (3). Family history-based testing for *BRCA1/2* germline mutations has been shown to miss a significant proportion of women at risk for developing cancer (5), perhaps as a result of incomplete or incorrect family history reporting (6, 7) or potentially due to a lack of updating new family history information as it becomes available (8).

At present, a variety of selection criteria are used to determine the eligibility for *BRCA1/2* testing, including family history, age at onset, tumor clinicopathological features, and computational risk prediction models (BRCAPRO, BOADICEA, Myriad, and Manchester scoring system) (9). The clinical criteria for risk assessment, genetic counseling, and genetic testing for *BRCA*-related cancers in women are based on personal and family history factors that may contribute to the disease (10) and are related to the likelihood of testing positive above a common testing threshold of 10% (11). These models often underestimate the probability of finding a mutation (12–14). It has been shown that the current family history approach does not identify 60% of Ashkenazi Jewish *BRCA* mutation carriers (15), thus creating a critical gap in practice that affects clinical treatment strategy and possibly patient outcome. As such, in light of advances in our understanding of *BRCA*-related breast and ovarian cancers – and the opportunity to directly impact therapeutic decision-making in these women – the recommendations to include universal germline *BRCA1/2* testing to all women diagnosed with non-mucinous ovarian carcinoma (4) and women with triple-negative breast cancer (16) are growing in strength (17–19). Using next generation sequencing for 21 tumor suppressor genes of 360 subjects, ~24% carried germline loss-of-function mutations: 18% in *BRCA1* or *BRCA2* and 6% in *BARD1, BRIP1, CHEK2, MRE11A, MSH6, NBN, PALB2, RAD50, RAD51C*, or *TP53* (20). The study also showed that 31% of women with an inherited mutation had no prior personal history of cancer or family history of breast or ovarian cancers (20). The National Comprehensive Cancer Network (NCCN) and Society of Gynecologic Oncology (SGO) guidelines suggest universal genetic counseling and testing of all women with ovarian cancer, including fallopian tube and peritoneal cancer (17, 19). Given the rate of *BRCA1/2* mutation in HGSOC, germline *BRCA1/2* testing is especially warranted in practice for this histology subtype. An immediate improvement to treatment opportunities would be to offer systematically genetic testing for *BRCA1/2* mutation to all HGSOC, although it has been reported that 20% of women with ovarian cancer in community hospital settings were referred for genetic testing (21). While this was shown to be improved in academic centers, referral for germline *BRCA1/2* testing was not systematic and did not reach the majority of patients (22). In clinical practice, there is a critical gap between the women eligible for *BRCA1/2* counseling and those receiving testing (23, 24). With the recent approval of olaparib, a PARP inhibitor, it is likely that referral for genetic testing of *BRCA1/2* status will improve.

## Knowledge of *BRCA1/2* Mutation Status Impacts Clinical Care of Women with Ovarian Cancer

Knowledge of *BRCA1/2* status should be part of the standard of care at least for patients diagnosed with HGSOC. Indeed, there is a large body of evidence indicating benefits of targeting pathways involved in maintaining DNA integrity, including *BRCA1* and *BRCA2* signaling (25). Harboring a germline *BRCA1/2* mutation is described as predictive of platinum sensitivity (26). Moreover, based on the synthetic lethality concept – the simultaneous promotion of *DNA* double-strand breaks (DSBs) and hindrance of DSB repair by inhibition of PARP protein expression (27, 28) – PARP inhibitors have been developed. This effect was shown clinically in the pivotal international, multicenter, randomized, phase II study that evaluated olaparib (a PARP inhibitor) as maintenance treatment in women with HGSOC who had responded to platinum-based chemotherapy (29). The preplanned retrospective analysis of outcomes by *BRCA1/2* status in this study demonstrated that *BRCA*-mutated patients had better progression-free survival (PFS) with olaparib maintenance compared to those receiving placebo (11·2 versus 4·3 months; HR 0·18; *p* < 0·0001) (30). The PFS benefit was still observed when somatic *BRCA*-mutated patients were included in the analysis. Additional evidence supporting the role of olaparib as maintenance therapy was reported from an international, multicenter, randomized, open-label study in women with platinum-sensitive relapsed HGSOC (NCT01081951) (31). In this phase II, olaparib was given with carboplatin/paclitaxel chemotherapy and continued as maintenance monotherapy. Overall, study findings show a significant PFS improvement when compared to chemotherapy alone (12.2 and 9.6 median PFS, respectively; HR 0.51; 95% CI 0.34–0.77; *p* = 0.0012). A greater benefit was detected in patients with a *BRCA1/2* mutation (PFS HR 0.21; 95% CI 0.08–0.55; *p* = 0.015) than in those without a *BRCA1/2* mutation. Further, study analysis revealed strong evidence that olaparib maintenance is most likely a key contributor to the improvement in PFS in this patient population (31). There are numerous ongoing PARP inhibitor studies investigating women with *BRCA1/2* mutations as well as mutations in other homologous recombination-deficient (HRD) genes, as data has shown HRD genes to exhibit *BRCA*-like behavior (32). To date, the use of olaparib maintenance has been approved in Europe after response to platinum-based chemotherapy in women with platinum-sensitive HGSOC who harbor a germline or somatic *BRCA1/2* mutation (30) and in US, as single agent therapy after three lines of chemotherapy in patients with germline *BRCA1/2* mutation HGSOC (33). Taken together, germline and somatic testing for *BRCA1/2* provides important information for patients with ovarian cancer and this knowledge can directly impact clinical care.

## Knowledge of Germline *BRCA1/2* Mutation Status Impacts Ovarian Cancer Prevention

Germline *BRCA1/2* status is not only relevant to women with ovarian cancer but also to women without cancer, who may be at an increased risk of developing the disease and could therefore benefit from prevention strategies. Currently, few prevention options are available for women with germline *BRCA1/2* mutations. Women known to be at an increased genetic risk for developing OC, based on germline *BRCA1/2* mutation carrier status, are offered risk-reducing salpingo–oophorectomy (RRSO), which reduces the risk of ovarian cancer by 71–96% (34–39). Surgery is usually performed after the completion of childbearing and while the woman is still pre-menopausal. Guidelines from the NCCN and the Society of Gynecologic Oncologists suggest that RRSO be completed by the age of 40 (19, 40); however, the majority of women who undergo RRSO do not do so by this age (41). This may be due to the potential side effects, such as premature surgical menopause (42), osteoporosis (43), cardiovascular disease (44, 45), cognitive impairments (46), symptoms of depression and anxiety (47), and consequences on quality of sleep, depression, and sexual dysfunction (48) associated with early RRSO. In light of these side effects – and the compelling evidence that high-grade serous epithelial ovarian cancer can be derived from the fallopian tube and not the ovary (49–53) – a recent committee opinion published by the American College of Obstetricians and Gynecologists outlines the opportunity for surgeon-led discussions with patients regarding the potential benefits of the removal of the fallopian tubes during hysterectomy in women at population risk of ovarian cancer who are not having an oophorectomy (54). Young *BRCA1/2* mutation carriers can be counseled for risk-reducing bilateral salpingectomy initially, with subsequent bilateral oophorectomy after childbearing, although additional randomized controlled trials are warranted to support the validity of this approach. Further studies of associated hysterectomy are warranted in the population to provide appropriate family counseling guidance (55, 56). These discussions are important as data from nine countries have shown that preventative practices in women with germline *BRCA1/2* mutations are varied despite guidelines (57). The study of 2677 women harboring germline *BRCA1*/*2* mutations, who were an average of 3.9 years following genetic testing, showed that only 57.2% had undergone prophylactic surgery. This reveals differing uptake of preventative options by their country of residence (57). It also highlights the lack of effective alternate strategies for individuals identified to be at high risk, often for years before clinical development of disease or risk reduction procedures like surgery can be offered.

## Germline *BRCA1/2* Testing Strategy

**Figure 2:**
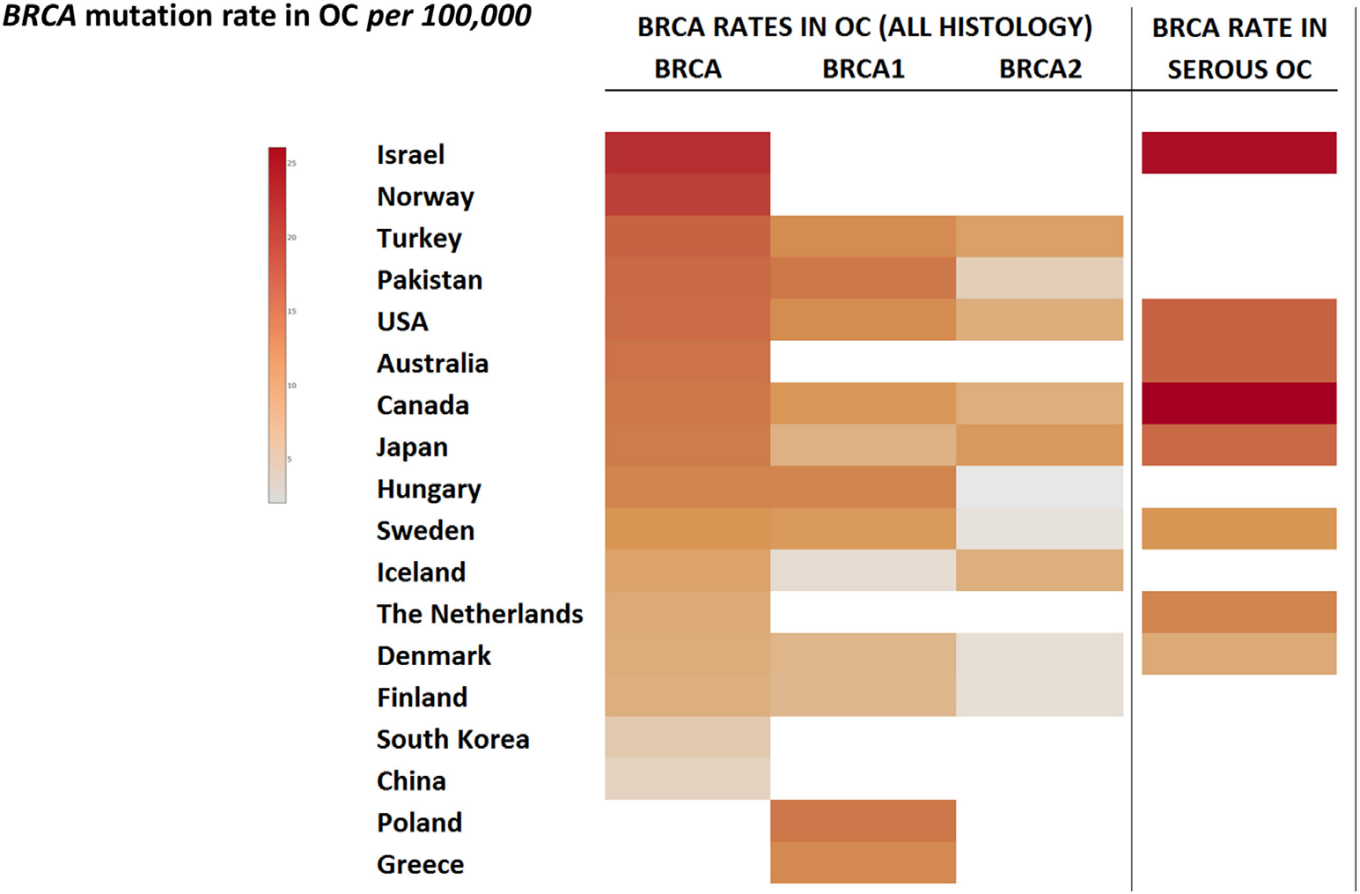
**Germline *BRCA1/2* mutational frequencies worldwide – examples**.

The current germline *BRCA1/2* testing strategy is mainly based on patients diagnosed with cancer. As previously discussed, as a minimum, all patients with HGSOC should be approached for *BRCA1/2* testing as well as those patients diagnosed with non-mucinous ovarian cancer (Figure 2). Furthermore, knowledge of germline *BRCA1/2* status in women living with ovarian cancer directly impacts first-degree relatives (FDRs), who have a 50% probability of carrying the same mutation and are yet to be diagnosed, and therefore, could also benefit from risk-reducing prevention strategies (58).

While there has been much debate regarding the concept of population-based germline *BRCA1/2* screening (59), this targeted approach within the Ashkenazi Jewish community has been shown to be more effective than family history-based testing and cost-effective. A Canadian-led study comparing the detection of *BRCA1/2* mutation carriers through Jewish population-based genetic testing versus clinic-based genetic testing found that more unaffected women with a *BRCA1* or *2* mutation were identified as a result of a genetic testing program targeting all Jewish women (60). This evidence supports the provision of genetic testing to all Jewish women (60). Conducted between 2008 and 2012, around 6179 Jewish women were tested through the population-based program, which identified 93 mutation carriers (92 unaffected with cancer) in comparison to 38 female carriers identified through 487 referrals to the genetics center (29 unaffected with cancer). Study findings showed that population genetic testing does not contribute to increased genetic counseling time but in fact decreases the overall time required when utilizing a population-based approach. Of particular importance, the 38% of women identified as having a *BRCA1/2* mutation would have qualified for genetic testing but were either unaware of the recommendation or had not been referred by their health-care provider (60). Examining a similar approach, a randomized controlled trial of germline *BRCA1/2* gene mutation testing in Ashkenazi Jewish women that compared family based testing to population screening, successfully enrolled and randomized 1034 participants (691 women, 343 men), of which 1017 were eligible for analysis. Similarly, findings showed that overall 56% of carriers did not fulfill clinical criteria for genetic testing, and germline *BRCA1/2* prevalence was 2.45%. The fact that more than half of participants did not fulfill testing criteria is in agreement with previous data (61, 62), in which carriers lacked a strong family history of cancer. Moreover, the study also provided evidence that population-based genetic testing of Ashkenazi Jewish women does not adversely affect short-term psychological or quality of life outcomes (63). Cost-effective analyses conducted in parallel to the above study show that even when incorporating *BRCA1/2* prevalence in family history negative individuals and genetic counseling costs, this specific population-based screening for germline *BRCA1/2* mutations is highly cost-effective compared to family history-based approaches in Ashkenazi Jewish women aged 30 years and older (15). Screening based on founder mutations is feasible if the type of mutation is well known and allows for population-based screening approaches, such as in the Ashkenazi Jewish population, where two founder mutations in *BRCA1* (185delAG and 5382insC) and one in *BRCA2* (6174delT) account for 98–99% of identified mutations (64–67). This population-based screening approach is cost-effective, as previously described, given that 2.5% of this population carry one of these three mutations (64), and these mutations account for 40% of ovarian cancer (68, 69).

**Figure 3:**
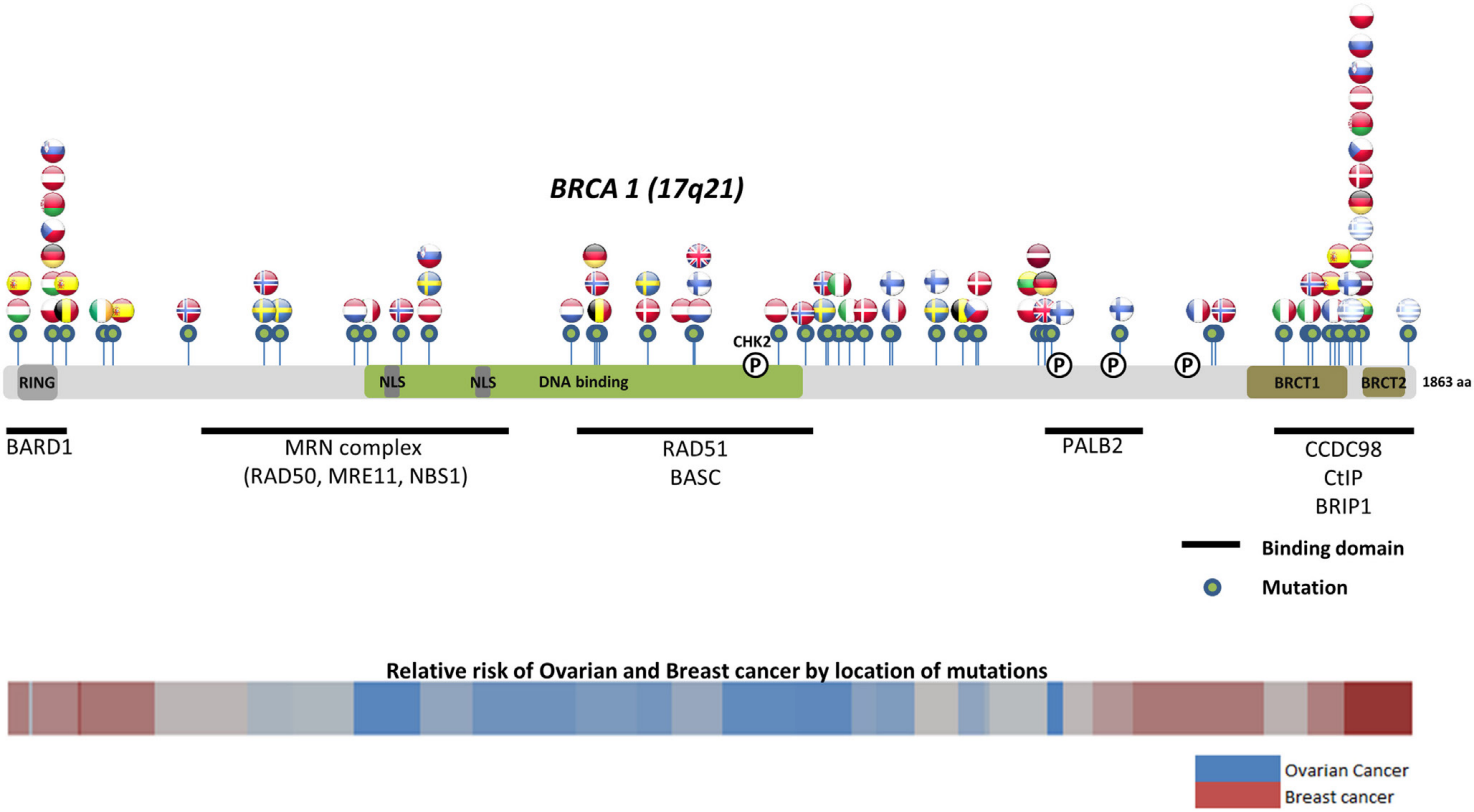
**Location of common and founder *BRCA1* mutations by country**. Schematic of common and founder mutations by country modified from Janavičius (71) and Ramus and Gayther (72). Relative risk data taken from Rebbeck et al. (73).

**Figure 4:**
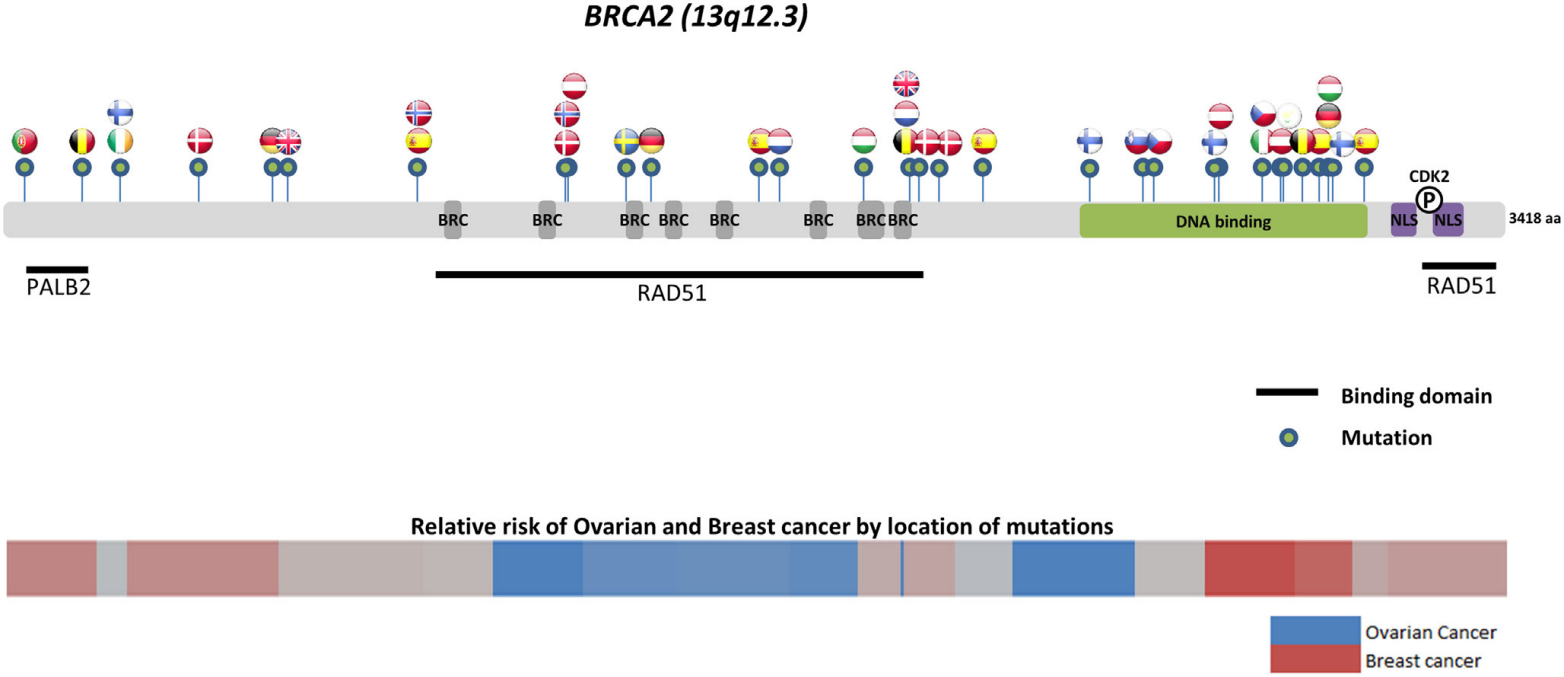
**Location of common and founder *BRCA2* mutations by country**. Schematic of common and founder mutations by country modified from Janavičius (71) and Ramus and Gayther (72). Relative risk data taken from Rebbeck et al. (73).

Worldwide, variation in the distribution of *BRCA1* and *BRCA2* mutations is well recognized, and in certain countries and ethnic communities the germline *BRCA1/2* mutation spectrum is limited to a few founder mutations (70). However, both the number and frequency of germline *BRCA1* and *BRCA2* mutations vary among populations (Figures 3 and 4) (71–73). Findings from an international observational study of 19,581 *BRCA1* and 11,900 *BRCA2* carriers from 55 centers in 33 countries on 6 continents provide strong evidence that breast and ovarian cancer risks vary by type and location of *BRCA1/2* mutation (73). As such, much research is moving toward characterizing the functional significance of specific mutations or mutation locations (74, 75).

Located on the long arm of chromosome 17, *BRCA1* (MIM#113705) comprises 22 coding exons spanning 80 kb of genomic DNA and has a 7.8-kb transcript coding for an 1863-amino-acid protein (76). *BRCA2* (MIM#600185) is located on chromosome 13 and comprises 26 coding exons spanning 70 kb of genomic DNA and gives an 11.4-kb transcript that encodes a protein of 3418 amino acids (77). Multifunctional in nature, *BRCA* proteins play important control functions in homologous recombination, the DNA DSB repair pathway, and early cellular response to DNA damage. *BRCA1* also has a transcriptional activator or repressor function and possesses a central role in chromatin remodeling and centrosome regulation. *BRCA1* and *BRCA2* appear to behave as tumor suppressor genes, and mutations in either of these genes have been found throughout the entire coding region and at splice sites (78). In light of the structural and interactive complexity of *BRCA1/2*, international collaborations will not only continue to improve our understanding of *BRCA1/2* mutations and how mutation type and location influence breast and ovarian cancer risks (Figures 3 and 4) (71–73) but also help devise novel, targeted testing panels that can potentially support specific population-based genetic testing, similar to the Ashkenazi Jewish population.

## Translating Knowledge into Practice

To ensure successful uptake of germline *BRCA1/2* testing or preventative strategies, wide community engagement and education regarding ovarian cancer are imperative. Following Angelina Jolie’s announcement that she carried a genetic mutation that increased her odds of developing breast and ovarian cancer, referrals for genetic counseling and *BRCA1/2* testing appeared to have increased the awareness of cancer, particularly for breast cancer (79–81). While celebrities can bring heightened awareness to health issues, there is a need for these messages to be accompanied by more purposeful communication efforts to assist the public in understanding and using the complex diagnostic and treatment information that these stories convey (82).

In a small US study, data show that despite a significant proportion of primary care patients requiring genetic counseling, there is compelling evidence that few are actually receiving these services (23). Data from the same study also indicate that while overall perceived cancer risk was higher among women with familial cancer risk, 27% of women with familial breast/ovarian cancer felt their risk was “low” and 32% felt their risk was lower than average – highlighting the need for educational interventions for patients as well as providers (23). This highlights the importance of considering the potential psychological impacts that may be associated with *BRCA1/2* testing over time. Employing qualitative interviews (*N* = 49) and reflective diaries, a study of 33 patients showed that the short-term impact of a positive *BRCA1/2* test result differs prior to, immediately following, and up to 24 months after having received test results (83). Conducted from December 2006 to March 2010, data show that while women with cancer initially undergo genetic testing for their children, on confirmation of a positive test, the focus temporarily shifts to decision-making around their personal health needs. In fact, the threat of further disease caused anxiety around nurturing children and personal survival, which remained unresolved until women underwent risk-reducing surgery and in many continued as cancer worry (83). Here, findings help to illustrate where additional support for women during the testing process may be most beneficial. The long-term effects of a positive *BRCA1/2* test result are also of relevance. A prospective single US centre study evaluating the long-term psychosocial effects of *BRCA1/2* testing in a cohort of 464 women who had undergone genetic testing found that at long-term follow up (median 5 years; range 3.4–9.1 years), when assessing cancer-specific and genetic testing distress, perceived stress, and perceived cancer risk, there is modest increased distress in *BRCA1/2* carriers compared to those women who received uninformative or negative test results (84). Despite the modest increase in distress, the group found no evidence of clinically significant dysfunction or impact of long-term psychological dysfunction due to testing (84). Data indicate that when patients receive counseling both before and after testing, they have more knowledge and experience less uncertainty and anxiety after learning the results of *BRCA1/2* test. Although, patient experiences may vary with test results (85). Therefore, when taken together, it is imperative that appropriate multidisciplinary, supportive structures are in place that women eligible for testing can rely upon, leading up to and following a positive test result, including at the time of risk-reducing surgery and during surveillance.

Testing positive for a germline *BRCA1/2* mutation goes beyond the patient herself potentially impacting her children and other members of their family by allowing cascade testing to proceed, if warranted (86). Accurate communication of test results is therefore critical for subsequent members to be tested. Research suggests fractured information dissemination among families when a positive germline *BRCA1/2* test is communicated. In a systematic review of 29 publications from 26 studies, family communication regarding genetic risk is described as a deliberative process whereby the individual’s personal risk is determined, within the context of family dynamics, family vulnerability and receptivity is assessed, which mediates what information will be conveyed, and ultimately, the appropriate time to disclose information (87). Numerous studies provide complementary data illustrating that issues impacting the communication of test results within families includes an individual’s responsibility to inform, emotional and developmental readiness – such as when parents disclose *BRCA1/2* results to children (88) – and again, communicating in the context of the existing family culture (89, 90). A retrospective study highlighted many errors in the transmission of DNA-test results in families from early stages of probands recalling information directly from genetic counselors, to the interpretation of information by family members (91). Therefore, support provided by genetic counselors could improve the overall process, not only during communication to family members but also during the education of physicians regarding family centered genetic testing for the physicians who may have referred the patient for testing (92).

## *BRCA1/2* Mutation Impacts More than Ovarian Cancer Treatment and Prevention

While the most described cancers driven by germline mutations in *BRCA1/2* have been breast and ovarian, there is also mounting evidence to support the role of germline *BRCA1/2* mutations contributing to other solid tumors, such as in prostate (93) and pancreatic (94, 95) cancers. In a United Kingdom study, Kote-Jarai et al. screened 1864 men with prostate cancer between 36 and 88 years of age and following analysis of the *BRCA2* gene, findings show that all carriers of truncating mutations developed prostate cancer at ≤65 years (93). In this study, the prevalence of *BRCA2* mutations was 1.27% (8/632) for cases diagnosed ≤55 years, 1.20% (19/1589) for cases diagnosed ≤65 years, and 0% (0/243) for cases diagnosed >65 years; *p* = 0.14 (81). It is estimated that germline mutations in the *BRCA2* gene confer an ~8.6× increased risk of prostate cancer by 65 years of age, corresponding to an absolute risk of ~15% by age 65. A higher risk is perhaps conferred due to mutations in the *BRCA2* ovarian cancer cluster region (OCCR) (96). Data suggest that routine testing of early onset prostate cancer cases for germline *BRCA2* mutations would further help refine the prevalence of risk associated with *BRCA2* mutations (93). A study examining other cancers in 268 *BRCA1* and 222 *BRCA2* families in the United Kingdom from 1975 to 2005 using person-years at risk analysis showed *BRCA2* mutation increased risks for pancreatic cancers (RR 4.1, 95% CI 1.9–7.8) and uveal melanoma (RR 99.4 95% CI 11.1–359.8). Study data also showed possible novel associations with upper gastrointestinal malignancies and *BRCA1* mutations, although this requires confirmation in future large prospective studies (96). Recently, a study provided evidence supporting current recommendations for hereditary breast and/or ovarian cancer screening of cancers other than breast and ovarian by the NCCN. In the study of 1072 patients who tested positive for a deleterious *BRCA1/2* mutation, 1177 cancers comprising 30 different cancer types were detected (97). Findings show that while individuals harboring *BRCA1* mutation did not have a significant increase in the development of cancers other than breast and ovarian, a trend in melanoma was observed. In addition, patients harboring a *BRCA2* mutation had a significantly higher number of observed cases compared to expected cases for pancreatic cancer (SIR 21.7, 95% CI = 13.1–34.0; *p* < 0.001) in both men and women and prostate cancer in men (SIR 4.9, 95% CI = 2.0–10.1; *p* < 0.002) (97). Taken together, germline *BRCA1/2* mutations bear significance in more than just breast and ovarian cancers. Future studies are warranted to provide evidence of access to *BRCA1/2* testing and counseling for these cancers as well.

## Conclusion

Worldwide, give the high incidence of ovarian cancer, the opportunity to identify *BRCA1/2* carriers at the time of their cancer diagnosis – and those at risk for developing disease – can impact therapeutic interventions. Therefore, it also provides compelling evidence to improve and standardize *BRCA1/2* testing practices. This becomes further punctuated when the opportunity to prevent or diagnose disease early in FDRs is also considered. In appropriate settings, population-based testing may be effective in identifying individuals at risk, who, with current criteria, would otherwise be missed. Future research should strive to build novel, targeted testing panels that will facilitate treatment/prevention-based decision-making. Therefore, it will be important to invest in resources and approaches that will change how ovarian cancer and other solid tumors with *BRCA1/2* involvement are managed and prevented, to improve the current paradigm of care.

## Author Contributions

KK wrote the draft manuscript and reviewed the article. JB did the figures and reviewed the article. VB and AO reviewed the manuscript. SL worked on the concept, the manuscript writing, and the review of the article.

## Conflict of Interest Statement

The authors declare that the research was conducted in the absence of any commercial or financial relationships that could be construed as a potential conflict of interest.
